# Supplementary figures and images for: Dr.seq2: A quality control and analysis pipeline for parallel single cell transcriptome and epigenome data
Source: PLoS One. 2017 Jul 3;12(7):e0180583. doi: 10.1371/journal.pone.0180583 (PMC5495495; doi:10.1371/journal.pone.0180583)

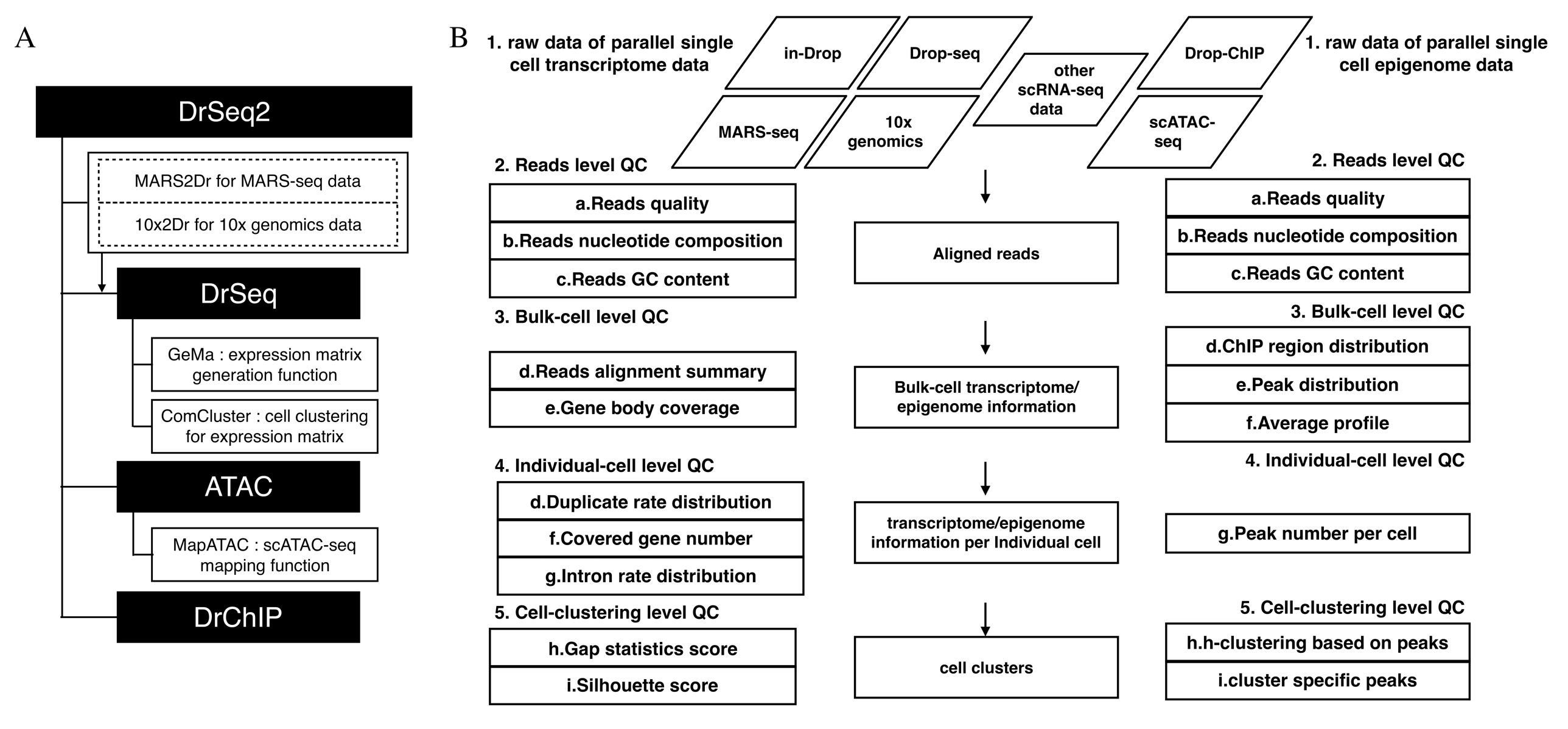

Supplement: S1 Fig — A) Dr.seq2 provides QC and analysis for three major data types: single cell transcriptome data (DrSeq part), Drop-ChIP data (DrChIP part) and scATAC-seq data (ATAC part). For single cell RNA-seq data, two additional step-by-step functions are included: 1. Expression matrix generation for amounts of single cell RNA-seq datasets (GeMa step) and 2. Cell clustering and analysis for the single cell expression matrix (comCluster step). For different parallel single cell RNA-seq technologies, input data are standardized for DrSeq part. B) Four groups of QC measurements are conducted on single cell transcriptome data and epigenome data: 1.Reads level QC including reads quality, reads nucleotide composition and reads GC content 2.Bulk-cell level QC including reads alignment summary and gene body coverage for transcriptome data; peak distribution; average profile on regulatory region and the distribution of different numbers of fragment length for epigenome data. 3. Individual-cell level QC including duplicate rate distribution, covered gene number and intron rate distribution and intron rate distribution for transcriptome data; peak number distribution and fragment length distribution for epigenome data. 4. Cell-clustering level QC including Gap statistics score and Silhouette score for transcriptome data, h-clustering and cluster specific peaks for epigenome data. (TIF) [file pone.0180583.s001.tif]

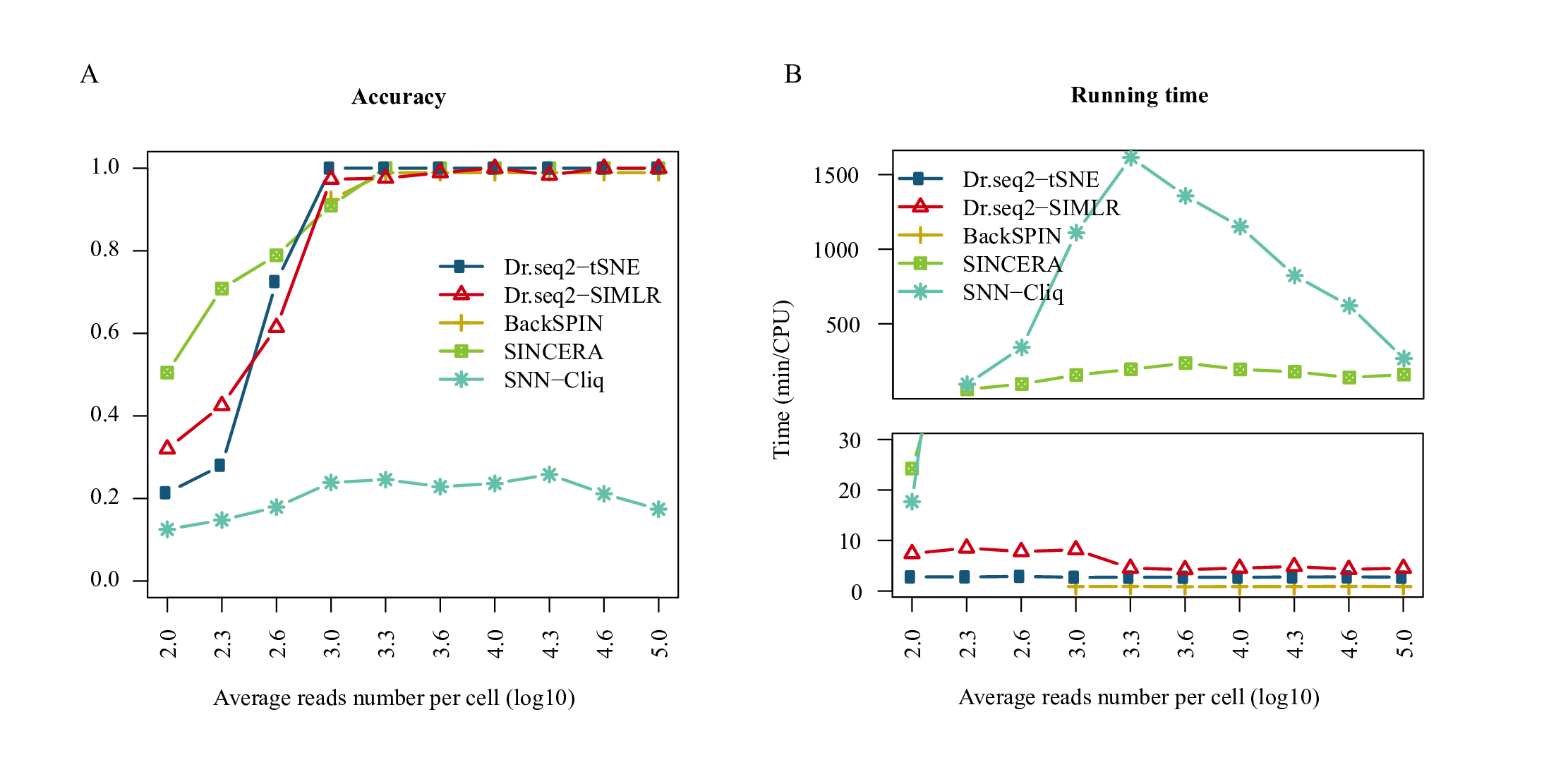

Supplement: S2 Fig — A) Clustering accuracy measured by the Goodman-Kruskal’s lambda index of Dr.seq2 t-SNE, Dr.seq2 SIMLR methods and three published methods on simulated data with different numbers of reads per cell. The lambda index (y-axis) is plotted as a function of the number of reads per cell (x-axis). B) Running time of Dr.seq2 t-SNE, Dr.seq2 SIMLR methods and three published methods on simulated data with different numbers of reads per cell. The running time (y-axis) is plotted as a function of the number of reads per cell (x-axis). The running time for each method was calculated using a single CPU (Intel® Xeon® CPU E5-2640 v2 @ 2.00 GHz). (TIF) [file pone.0180583.s002.tif]
