# Supplementary material for: Dr.seq2: A quality control and analysis pipeline for parallel single cell transcriptome and epigenome data
Source: PLoS One. 2017 Jul 3;12(7):e0180583. doi: 10.1371/journal.pone.0180583 (PMC5495495; doi:10.1371/journal.pone.0180583)
Supplement: S3 File — (PDF) [file pone.0180583.s005.pdf]

Dr.seq 2.0 QC and Analysis Summary Report: scATAC

May 19, 2017

Contents

1 Data description **2**

2 Bulk-cell level QC **3**
2.1 Reads alignment summary 4
2.2 Chromosomal Distribution of Open Regions 5
2.3 Peaks over Chromosomes 6
2.4 Average profile on different genome regions 7
2.5 Distribution of fragment numbers (excluded fragments in mitochondria) 8

3 Individual-cell level QC **9**
3.1 Distribution of peak numbers per cell 10

4 Cell-clustering level QC **11**
4.1 Cell clustering 12
4.2 Silhouette of clustering 13
4.3 Clustering heatmap 14
4.4 Ideogram 15

5 Output list **16**

1 Data description

Table 1 mainly describes the input file and mapping and analysis parameters.

Table 1: Data description

parameter	value
output name	scATAC
reads file (file name only)	SRR1779659.bam ...
fragment length threshold	1500
genome type	hs
p-value threshold for macs14	1e-5
height of cutting tree	0
given cluster numbers	3
limited number of peaks of informative cells	20
limited number of cells of informative peaks	20
Q30 filter mapped reads	True

2 Bulk-cell level QC

In the bulk-cell level QC step we measured the performance of total scATAC reads. In this step we did't separate reads, just like treated the sample as bulk ATAC-seq sample.

2.1 Reads alignment summary

The following table shows reads number after each filter strategy and mapped reads of final selected reads. It measures the general sequencing quality. Low mappability indicates poor sequence quality (see “Reads level QC”) or library quality (caused by contaminant).
In summary, if the percentage of “total mapped reads” is less than 5%, users may consider reconstruct your library (redo the experiment), but first you should make sure you already trim the adapter and map your reads to the corresponded species (genome version). Mappable reads was after Q30 filtering if Q30 filter function was turned on.

Table 2: Reads alignment summary

genomic region (Category)	reads number
total number of read	154,235,698
number of mapped reads	86,823,837 (56.29%)*
number of mitochondria reads	19,743,356 (12.8%)*

2.2 Chromosomal Distribution of Open Regions

The blue bars represent the percentages of the whole tiled or mappable regions in the chromosomes (genome background) and the red bars showed the percentages of the whole open region. These percentages are also marked right next to the bars. P-values for the significance of the relative enrichment of open regions with respect to the gnome background are shown in parentheses next to the percentages of the red bars.

Figure 1: Chromosomal Distribution of Open Regions

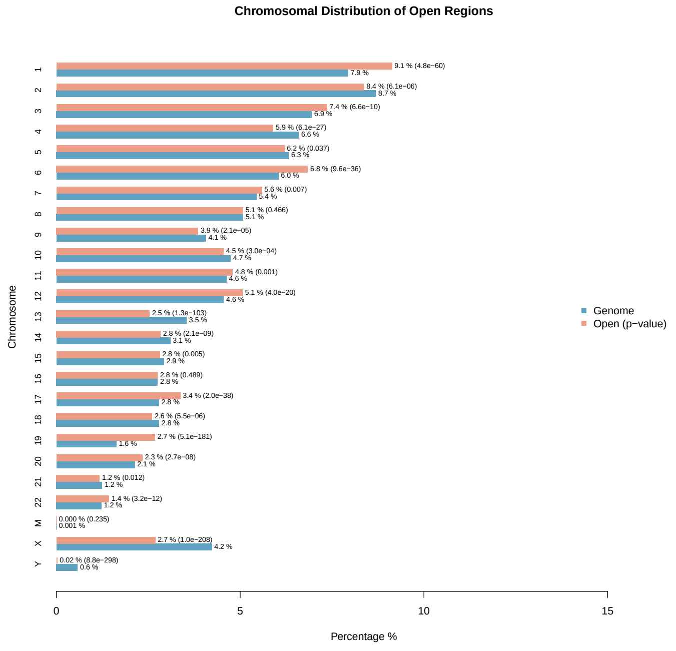

2.3 Peaks over Chromosomes

Barplot show open regions distributed over the genome along with their scores or peak heights. The line graph on the top left corner illustrates the distribution of peak heights (or scores). The red bars in the main plot open regions in the input BED file. The x-axis of the main plot represents the actual chromosome sizes.

Figure 2: Peaks over Chromosomes

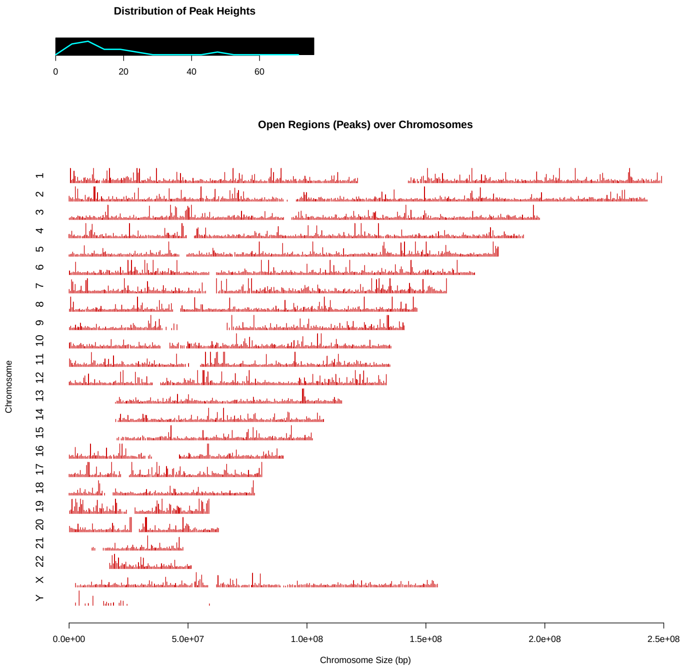

2.4 Average profile on different genome regions

Average profiling within/near important genomic features. The panels on the first row display the average enrichment signals around TSS and TTS of genes, respectively. The bottom panel represents the average signals on the meta-gene of 5 kb.

Figure 3: verage profile on different genome regions

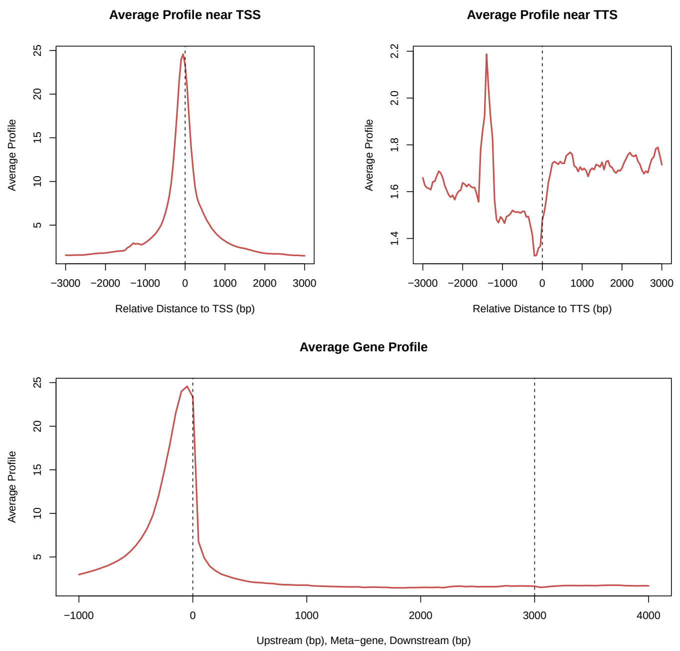

2.5 Distribution of fragment numbers (excluded fragments in mitochondria)

ATAC-seq indicated factor occupancy and nucleosome positions with periodicity fragment length distribution.

Figure 4: Distribution of fragment numbers (excluded fragments in mitochondria)

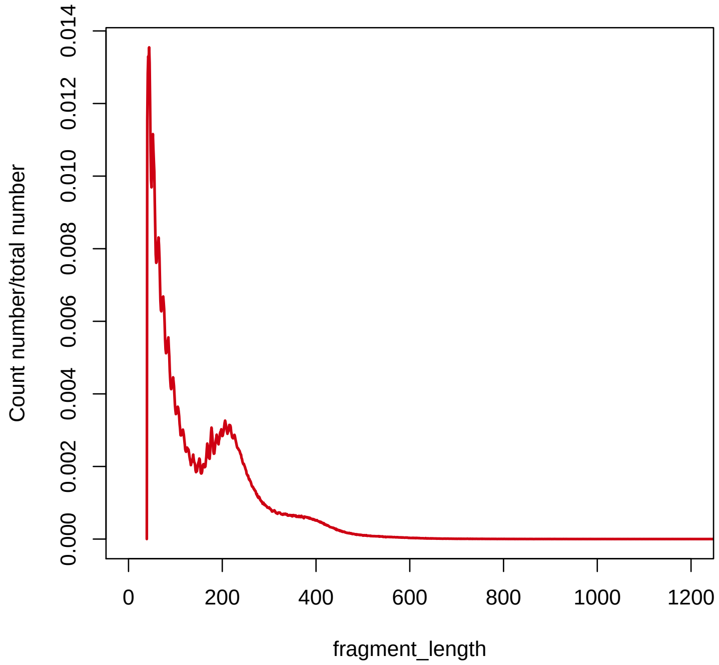

3 Individual-cell level QC

In this step we focused on the quality of individual cell by calculate the number of peak per cell overlapped with combined peaks

3.1 Distribution of peak numbers per cell

To measure whether the cell is informative for post-analysis, peak number per each cell is calculated, The cells with small number of peaks indicated the limited informative of cells.

Figure 5: Peak distribution

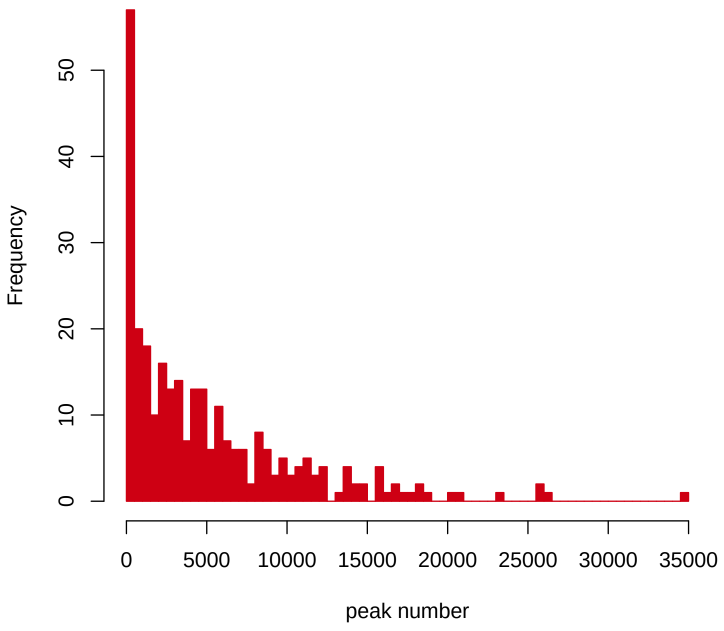

4 Cell-clustering level QC

This step composed by h-clustering based on macs14 peaks.

4.1 Cell clustering

We conducted a h-cluster based on macs14 peaks to measure sample's ability to be separated to different cell subtypes.

Figure 6: h-clustering based on peak

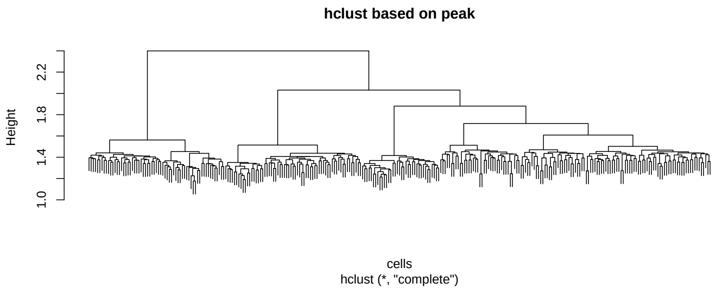

4.2 Silhouette of clustering

Silhouette method is used to interprate and validate the consistency within clusters defined in previous steps. A poor Silhouette (e.g. average si < 0.2) score indicate that the experiments (if not properly done) may not separate well the subpopulations of cells. If most of your clusters have poor Silhouette score, it may indicate a poor quality of your experiments.

Figure 7: Silhouette score for clustered STAMPs

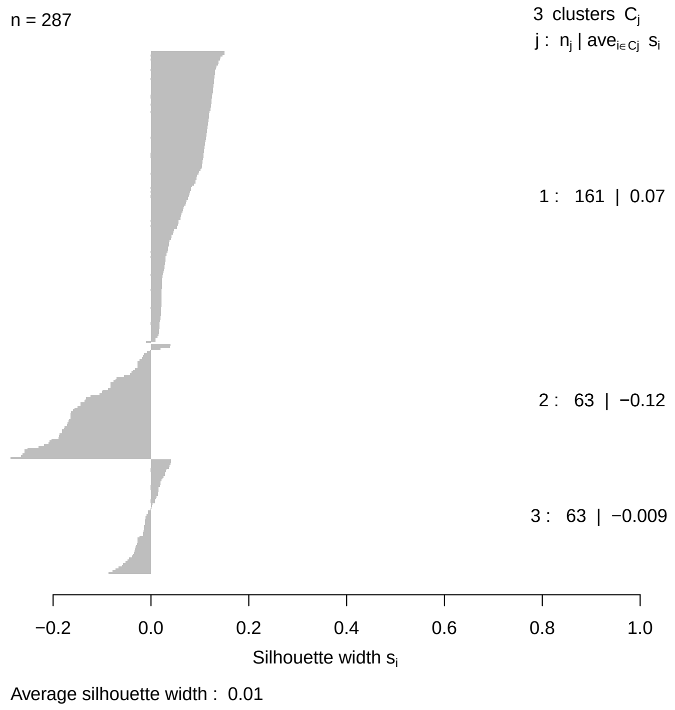

4.3 Clustering heatmap

Cell Clustering tree and peak region in each cell. The upper panel represents the hieratical clustering results based on each single cell. The second panel with different colors represents decision of cell clustering. The bottom two panels (heatmap and color bar) represent the "combined peaks" occupancy of each single cell.

Figure 8: h-clustering heatmap

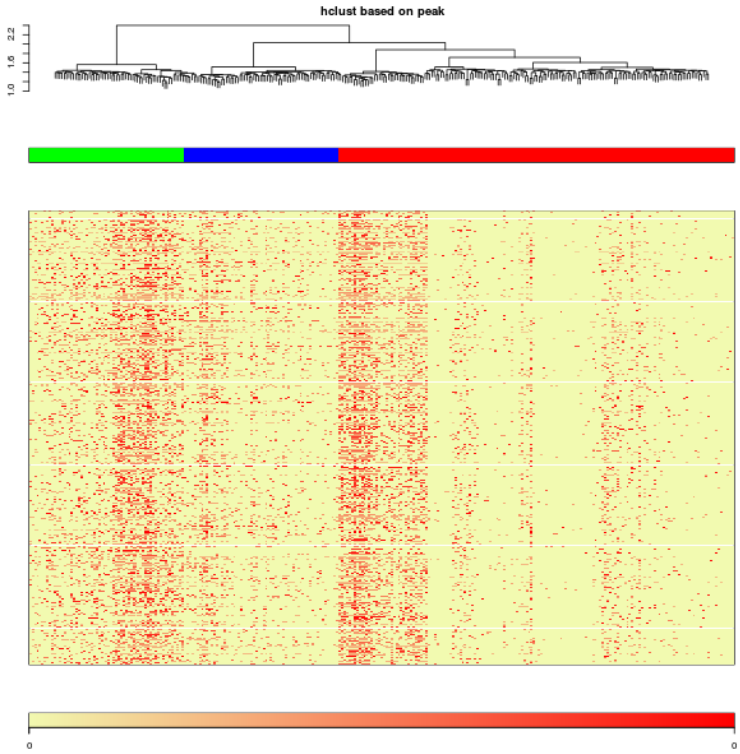

4.4 Ideogram

Cluster specific regions were show in each chromsome.

Figure 9: Ideogram of cluster specific regions

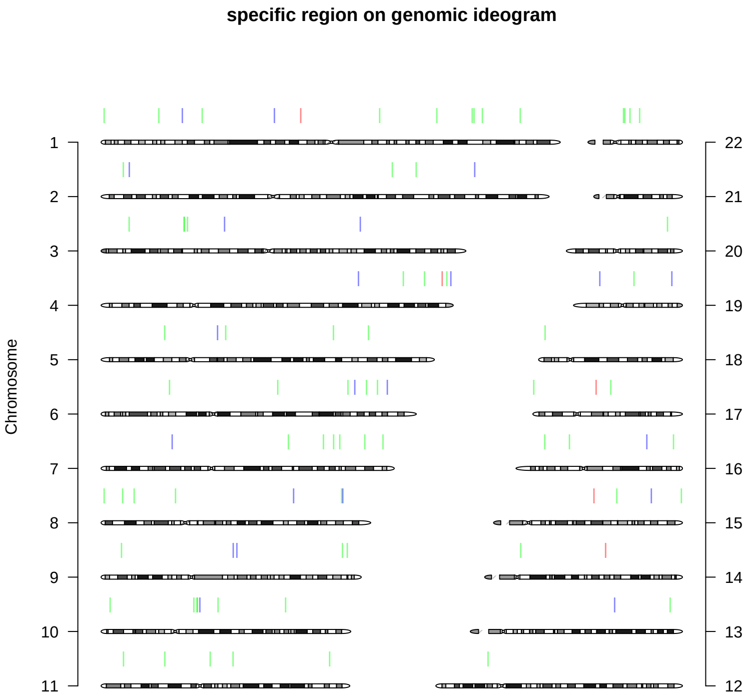

5 Output list

All output files were described in the following table

Table 3: output list

description	filename
peak location matrix for each cell	scATACpeakMatrix.txt
cells in each cluster	scATAC_cluster*_cells.txt
cell type specific peaks per each cluster	scATAC*_specific.peak.bed
cell clustering results with Silhouette Score	scATAC_cluster_with_silhouette_score.txt
combined peaks	scATAC_peaks.bed
summary QC report	scATAC_summary.pdf
