# Supplementary material for: Dr.seq2: A quality control and analysis pipeline for parallel single cell transcriptome and epigenome data
Source: PLoS One. 2017 Jul 3;12(7):e0180583. doi: 10.1371/journal.pone.0180583 (PMC5495495; doi:10.1371/journal.pone.0180583)
Supplement: S4 File — (PDF) [file pone.0180583.s006.pdf]

Dr.seq 2.0 QC and Analysis Summary Report: H3K4me2_140226_07

May 19, 2017

Contents

1 Data description **2**

2 Reads level QC **3**
2.1 Reads quality 4
2.2 Reads nucleotide composition 5
2.3 Reads GC content 6

3 Bulk-cell level QC **7**
3.1 Reads alignment summary 8
3.2 Chromosomal Distribution of ChIP Regions 9
3.3 Peaks over Chromosomes 10
3.4 average profile on different genome regions 11

4 Individual-cell level QC **12**
4.1 Reads distribution 13

5 Cell-clustering level QC **14**
5.1 Cell clustering 15
5.2 Silhouette of clustering 16
5.3 Clustering heatmap 17
5.4 Ideogram 18

6 Output list **19**

1 Data description

Table 1 mainly describes the input file and mapping and analysis parameters.

Table 1: Data description

parameter	value
output name	H3K4me2_140226_07
barcode file (file name only)	H3K4me2_140226_07selected_1.fastq
reads file (file name only)	H3K4me2_140226_07selected_2.fastq
reads file format	FASTQ
cell barcode range in read1	1:8
cell barcode range in read2	12:19
mapping software	bowtie2
Q30 filter mapped reads	True
maximum fragment length	1000
trim bases from 5'/left end of reads	23
threshold for macs14 peak calling	1e-4
size of peak extension	0
the max number of input barcode	1152

2 Reads level QC

In the reads level QC step we measured the quality of sequencing reads, including nucleotide quality and composition. In the reads level QC step and Bulk-cell level QC step we randomly sampled down total reads to 5 million and used a published package called “RseQC” for reference. (Wang, L., Wang, S. and Li, W. (2012))

2.1 Reads quality

Reads quality is one of the basic reads level quality control methods. We plotted the distribution of a widely used Phred Quality Score at every position of sequence to measure the basic sequence quality of your data. Phred Quality Score was calculate by a python function $ord(Q) - 33$. Color in the heatmap represented frequency of this quality score observed at this position. Red represented higher frequency while blue was lower frequency. You may observe a decreasing of quality near the 3'end of sequence because of general degradation of quality over the duration of long runs. If the decreasing of quality influence the mappability (see "Bulk-cell level QC") then the common remedy is to perform quality trimming where reads are truncated based on their average quality or you can trim serveal base pair near 3'end directly. If it doesn't help, you may consider your Drop-ChIP data poor quality.

Figure 1: Reads quality

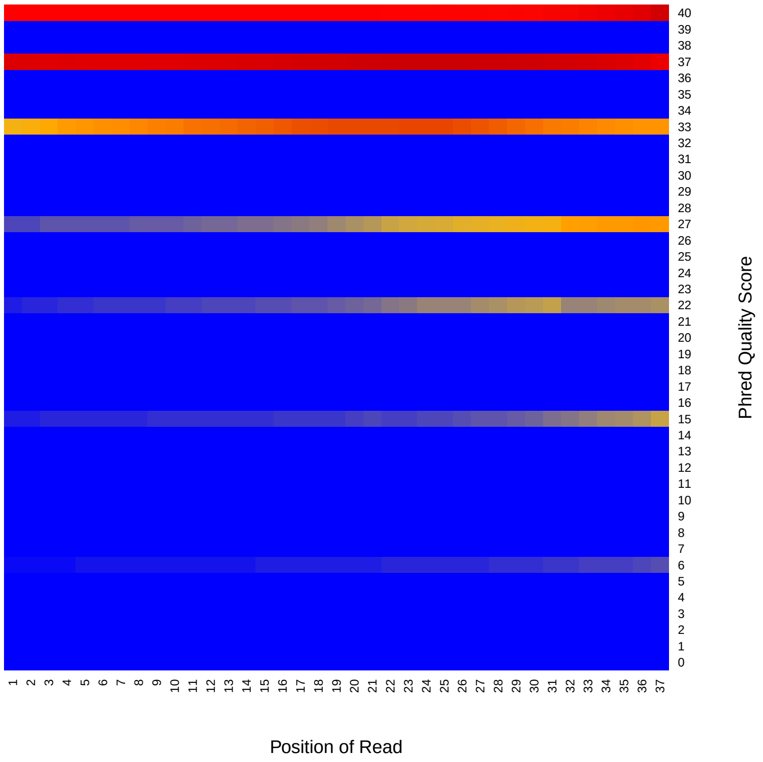

2.2 Reads nucleotide composition

We assess the nucleotide composition bias of a sample. The proportion of four different nucleotides was calculated at each position of reads. Theoretically four nucleotides had similar proportion at each position of reads. You may observe higher A/T count at 3'end of reads because of the 3'end polyA tail generated in sequencing cDNA libaray, otherwise the A/T count should be closer to C/G count. In any case, you should observe a stable pattern at least in the 3'end of reads. Spikes (un-stable pattern) which occur in the middle or tail of the reads indicate low sequence quality. You can trim serveral un-stable bases from the 3'end if low mappability (see "Bulk-cell level QC") is also observed. If it doesn't help, you may consider your Drop-ChIP data poor quality. Note that t he A/T vs G/C content can greatly vary from species to species.

Figure 2: Reads nucleotide composition

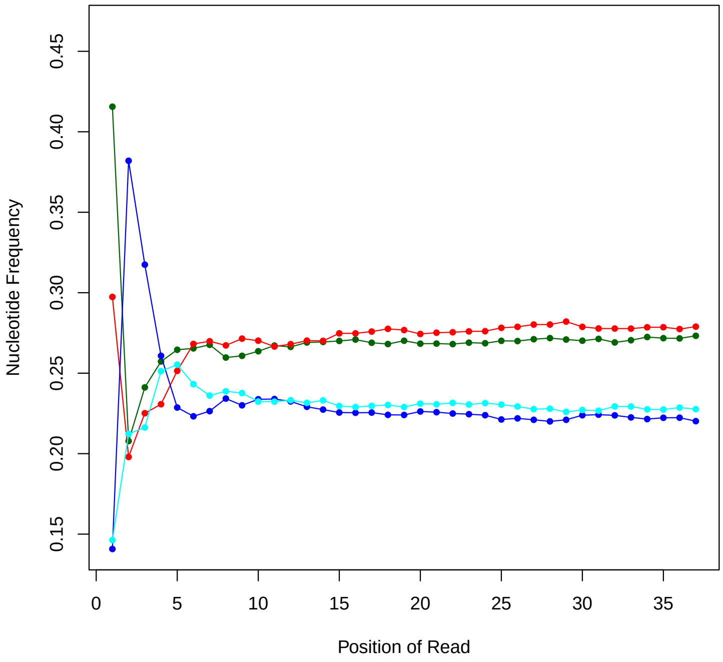

2.3 Reads GC content

Distribution of GC content of each read. This module measures the general quality of the library. If the distribution looks different from a single bell (too sharp or too broad) then there may be a problem with the library. Sharp peaks on an otherwise smooth distribution are normally the result of a specific contaminant (adapter dimers for example), which may well be picked up by the overrepresented sequences module. Broader peaks may represent contamination with a different species. If you observe sharp peak or broder peak and also observe low mappability (see "Bulk-cell level QC"), you may consider your Drop-ChIP data poor quality.

Figure 3: Reads GC content

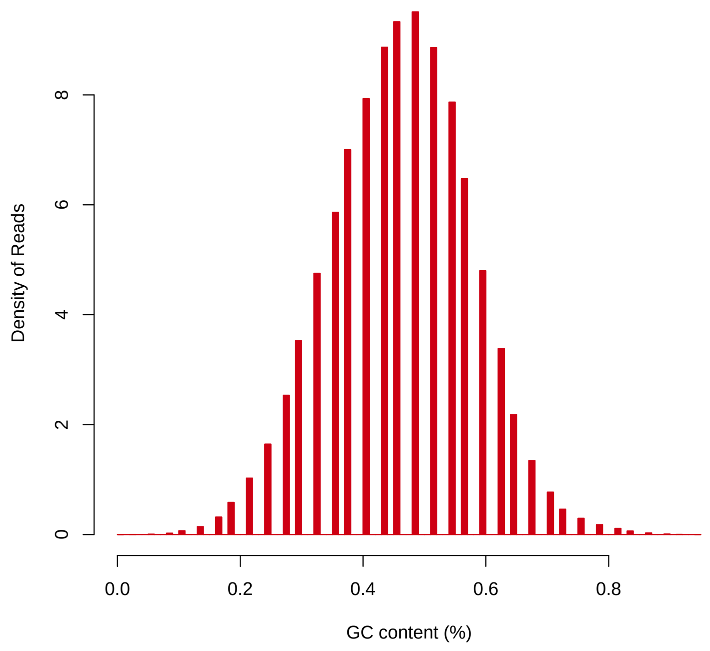

3 Bulk-cell level QC

In the bulk-cell level QC step we measured the performance of total Drop-ChIP reads. In this step we did't separate reads, just like treated the sample as bulk ChIP-seq sample.

3.1 Reads alignment summary

The following table shows reads number after each filter strategy and mapped reads of final selected reads. It measures the general sequencing quality. Low mappability indicates poor sequence quality (see "Reads level QC") or library quality (caused by contaminant). In summary, if the percentage of "total mapped reads" is less than 5%, users may consider reconstruct your library (redo the experiment), but first you should make sure you already trim the adapter and map your reads to the corresponded species (genome version). Mappable reads was after Q30 filtering if Q30 filter function was turned on.

Table 2: Reads alignment summary

genomic region (Category)	reads number
total number of read pairs	3,137,669
number of reads pairs after barcode filtered	430,993 (13.74%)*
number of mapped reads	371,191 (11.83%)*
number of reads after length filtered	371,180 (11.83%)*

3.2 Chromosomal Distribution of ChIP Regions

The blue bars represent the percentages of the whole tiled or mappable regions in the chromosomes (genome background) and the red bars showed the percentages of the whole ChIP. These percentages are also marked right next to the bars. P-values for the significance of the relative enrichment of ChIP regions with respect to the gnome background are shown in parentheses next to the percentages of the red bars.

Figure 4: Chromosomal Distribution of ChIP Regions

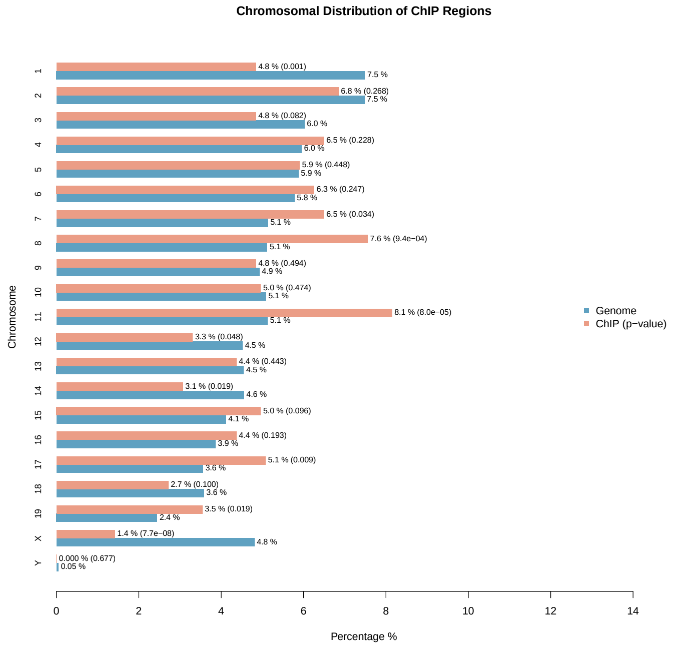

3.3 Peaks over Chromosomes

Barplot show ChIP regions distributed over the genome along with their scores or peak heights. The line graph on the top left corner illustrates the distribution of peak heights (or scores). The red bars in the main plot ChIP regions in the input BED file. The x-axis of the main plot represents the actual chromosome sizes.

Figure 5: Peaks over Chromosomes

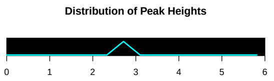

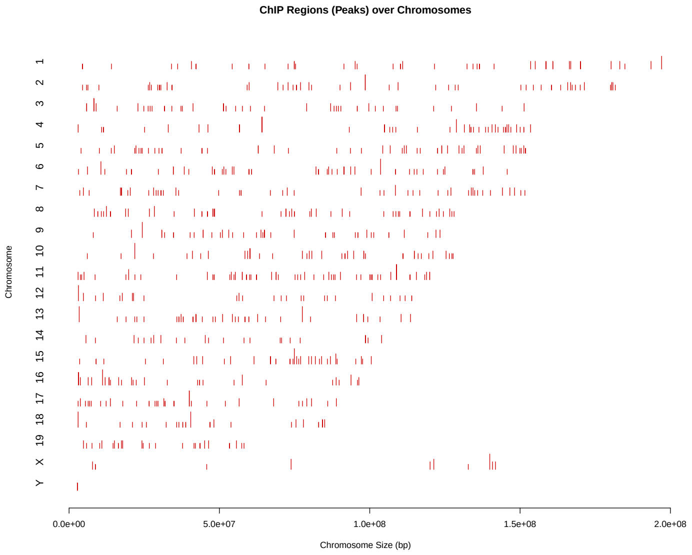

3.4 average profile on different genome regions

Average profiling within/near important genomic features. The panels on the first row display the average ChIP enrichment signals around TSS and TTS of genes, respectively. The bottom panel represents the average ChIP signals on the meta-gene of 5 kb.

Figure 6: average profile on different genome regions

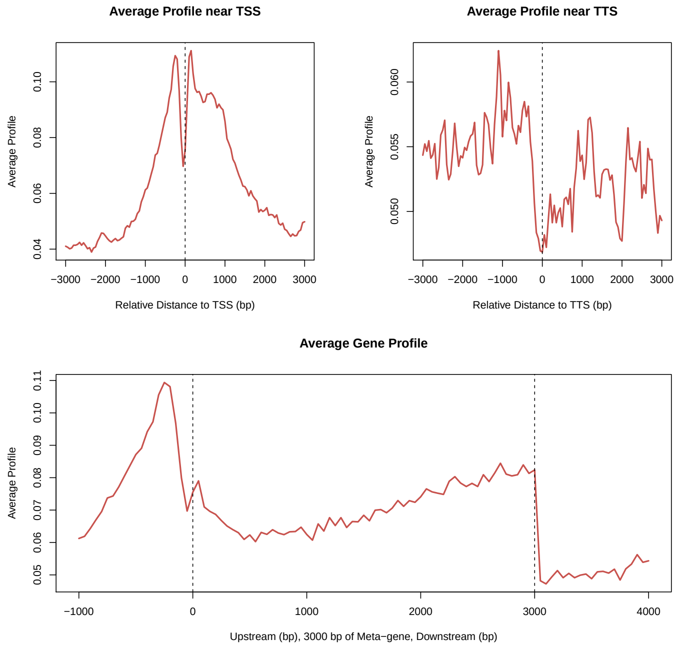

4 Individual-cell level QC

In this step we focused on the quality of individual cell and distinguishing cell reads from background reads

4.1 Reads distribution

Drop-ChIP technology has an innate advantage of detecting individual cell reads and background reads due to the barcode information. This module displays the distribution of reads number in each cell and helps to discard barcodes with high rate of background reads (which usually caused by empty cell barcodes and ambient sequence). We plot the distribution of reads number in each cell barcode (though most of cell barcodes don't contain cells, they still have reads) and observed a bimodal distribution of reads number. The red line show the reads distribution.

Figure 7: Reads distribution

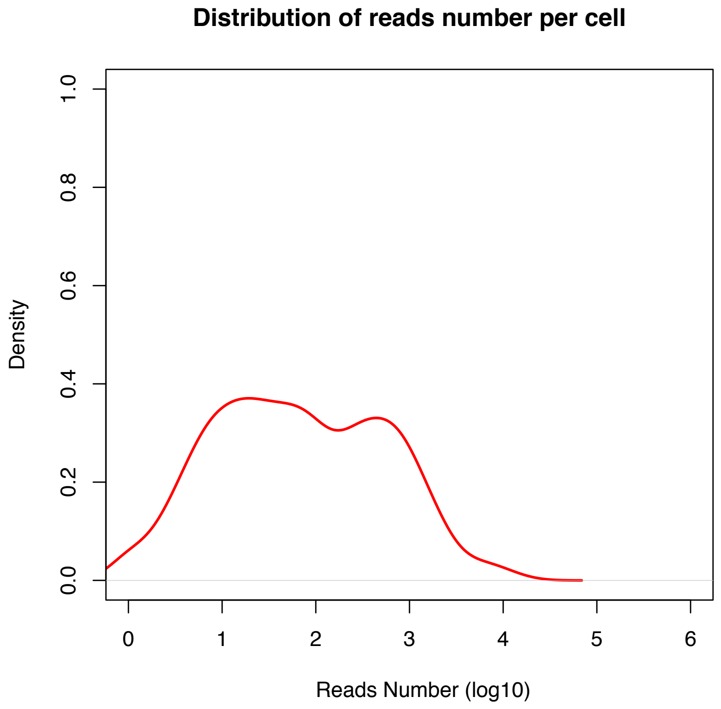

5 Cell-clustering level QC

This step composed by h-clustering based on macs14 peaks.

5.1 Cell clustering

We conducted a h-cluster based on macs14 peaks to measure sample's ability to be separated to different cell subtypes.

Figure 8: h-clustering based on peak

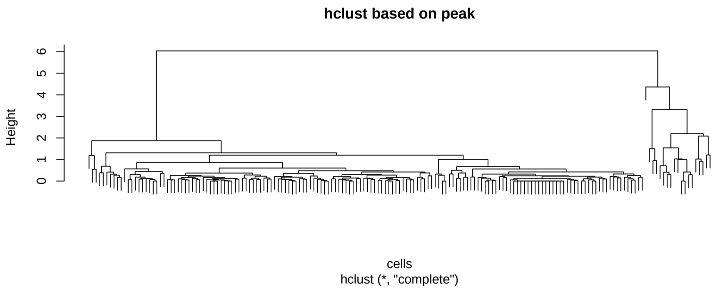

5.2 Silhouette of clustering

Silhouette method is used to interprate and validate the consistency within clusters defined in previous steps. A poor Silhouette (e.g. average si < 0.2) score indicate that the experiments (if not properly done) may not separate well the subpopulations of cells. If most of your clusters have poor Silhouette score, it may indicate a poor quality of your experiments.

Figure 9: Silhouette score for clustered STAMPs

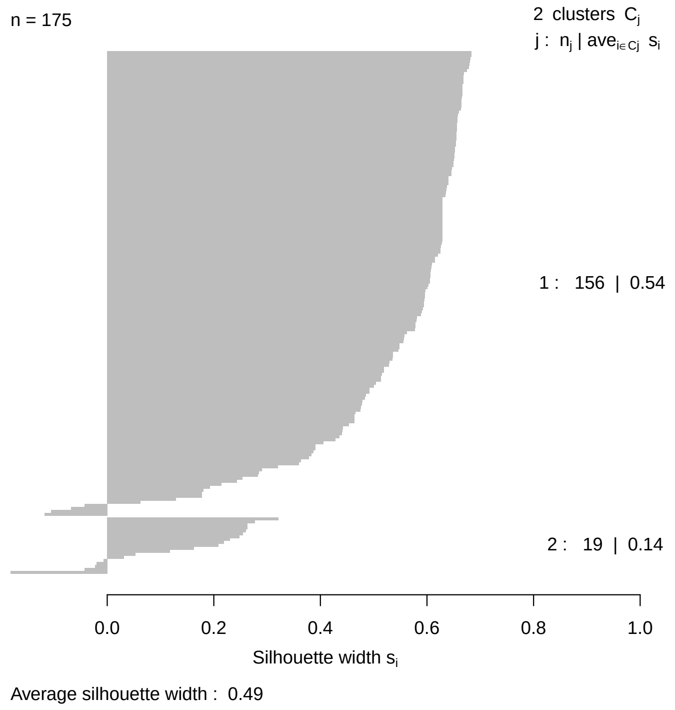

5.3 Clustering heatmap

Cell Clustering tree and peak region in each cell. The upper panel represents the hieratical clustering results based on each single cell. The second panel with different colors represents decision of cell clustering. The bottom two panels (heatmap and color bar) represent the "combined peaks" occupancy of each single cell.

Figure 10: h-clustering heatmap

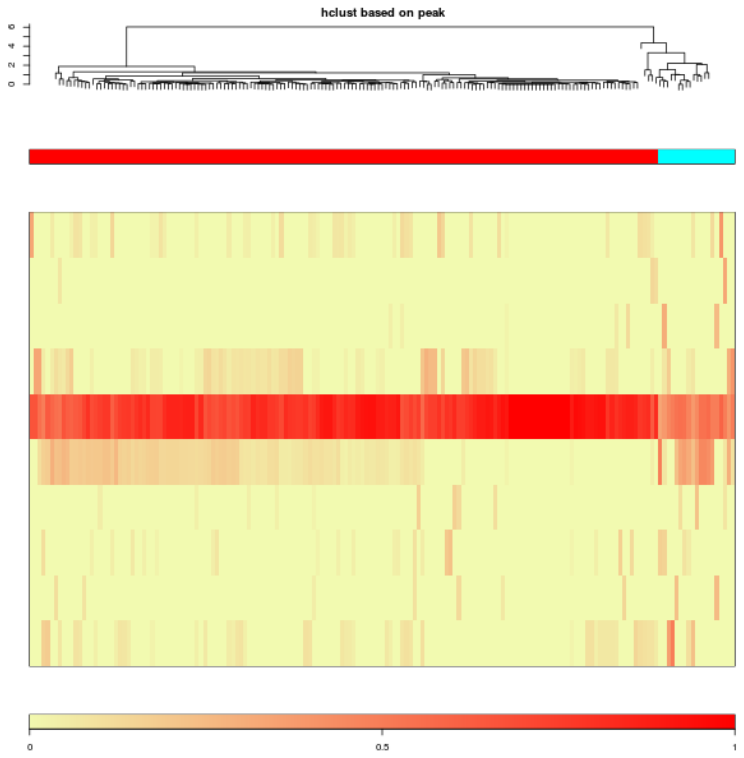

5.4 Ideogram

Cluster specific regions were show in each chromsome.

Figure 11: Ideogram of cluster specific regions

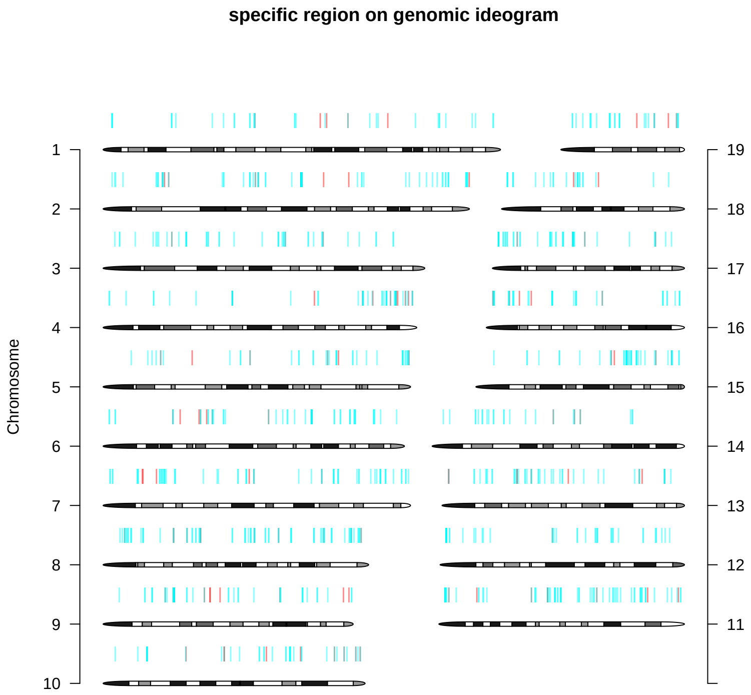

6 Output list

All output files were described in the following table

Table 3: output list

description	filename
peak location matrix for each cell	H3K4me2_140226_07_signal.txt
cells in each cluster	H3K4me2_140226_07_cluster*_cells.txt
cell type specific peaks per each cluster	H3K4me2_140226_07*_specific.peak.bed
cell clustering results with Silhouette Score	H3K4me2_140226_07_cluster_with_silhouette_score.txt
combined peaks	H3K4me2_140226_07_peaks.bed
summary QC report	H3K4me2_140226_07_summary.pdf
