# Supplementary material for: Dr.seq2: A quality control and analysis pipeline for parallel single cell transcriptome and epigenome data
Source: PLoS One. 2017 Jul 3;12(7):e0180583. doi: 10.1371/journal.pone.0180583 (PMC5495495; doi:10.1371/journal.pone.0180583)
Supplement: S5 File — (PDF) [file pone.0180583.s007.pdf]

Dr.seq 2.0 QC and Analysis Summary Report: jurkat_293t_50_50

May 19, 2017

Contents

1 Data description 2

2 Reads level QC 3
- 2.1 Reads quality 4
- 2.2 Reads nucleotide composition 5
- 2.3 Reads GC content 6

3 Bulk-cell level QC 7
- 3.1 Reads alignment summary 8
- 3.2 Gene body coverage 9

4 Individual-cell level QC 10
- 4.1 Reads duplicate rate distribution 11
- 4.2 Reads duplicate rate vs. cumulative covered gene number . . 12
- 4.3 UMI vs. covered gene number 13
- 4.4 Covered gene number distribution 14
- 4.5 Intron rate distribution 15

5 Cell-clustering level QC 16
- 5.1 Gap statistics 17
- 5.2 Clustering plot 18

6 Output list 19

1 Data description

Table 1 mainly describes the input file and mapping and analysis parameters.

Table 1: Data description

parameter	value
output name	jurkat_293t_50_50
barcode file (file name only)	jurkat_293t_50_50_1.fastq
reads file (file name only)	jurkat_293t_50_50.sam
reads file format	SAM
cell barcode range	1:22
UMI range	23:33
mapping software	STAR
Q30 filter mapped reads	True
remove reads away TTS	False
duplicate rate in each cell	UMI + location
merge UMI ED = 1	False
select STAMPs	1000 covered gene
remove low duplicate rate cell	False
z-score for highly variable gene	1.64
cumulative variance for selecting PC	50.0%
cluster method	k-means (Gap statistics, first stable)

2 Reads level QC

In the reads level QC step we measured the quality of sequencing reads, including nucleotide quality and composition. In the reads level QC step and Bulk-cell level QC step we randomly sampled down total reads to 5 million and used a published package called “RseQC” for reference. (Wang, L., Wang, S. and Li, W. (2012))

2.1 Reads quality

Reads quality is one of the basic reads level quality control methods. We plotted the distribution of a widely used Phred Quality Score at every position of sequence to measure the basic sequence quality of your data. Phred Quality Score was calculate by a python function $ord(Q) - 33$. Color in the heatmap represented frequency of this quality score observed at this position. Red represented higher frequency while blue was lower frequency. You may observe a decreasing of quality near the 3'end of sequence because of general degradation of quality over the duration of long runs. If the decreasing of quality influence the mappability (see "Bulk-cell level QC") then the common remedy is to perform quality trimming where reads are truncated based on their average quality or you can trim serveal base pair near 3'end directly. If it doesn't help, you may consider your Drop-seq data poor quality.

Figure 1: Reads quality

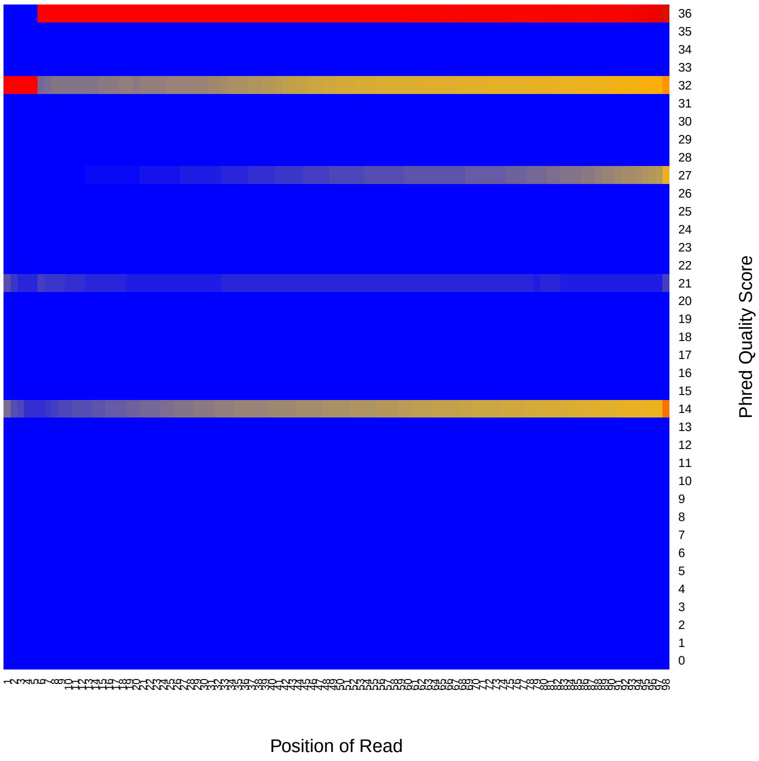

2.2 Reads nucleotide composition

We assess the nucleotide composition bias of a sample. The proportion of four different nucleotides was calculated at each position of reads. Theoretically four nucleotides had similar proportion at each position of reads. You may observe higher A/T count at 3'end of reads because of the 3'end polyA tail generated in sequencing cDNA libaray, otherwise the A/T count should be closer to C/G count. In any case, you should observe a stable pattern at least in the 3'end of reads. Spikes (un-stable pattern) which occur in the middle or tail of the reads indicate low sequence quality. You can trim serveral un-stable bases from the 3'end if low mappability (see "Bulk-cell level QC") is also observed. If it doesn't help, you may consider your Drop-seq data poor quality. Note that t he A/T vs G/C content can greatly vary from Getecies to Getecies.

Figure 2: Reads nucleotide composition

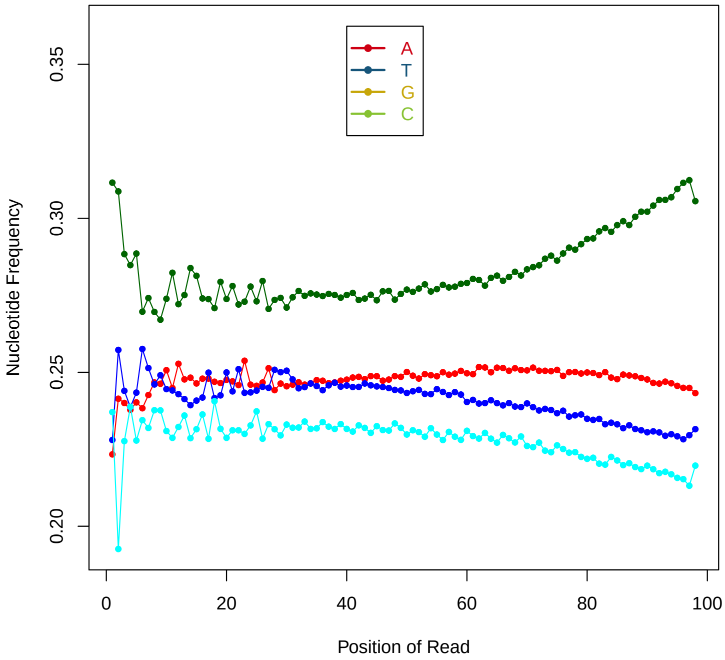

2.3 Reads GC content

Distribution of GC content of each read. This module measures the general quality of the library. If the distribution looks different from a single bell (too sharp or too broad) then there may be a problem with the library. Sharp peaks on an otherwise smooth distribution are normally the result of a Getecific contaminant (adapter dimers for example), which may well be picked up by the overrepresented sequences module. Broader peaks may represent contamination with a different Getecies. If you observe sharp peak or broder peak and also observe low mappability (see "Bulk-cell level QC"), you may consider your Drop-seq data poor quality.

Figure 3: Reads GC content

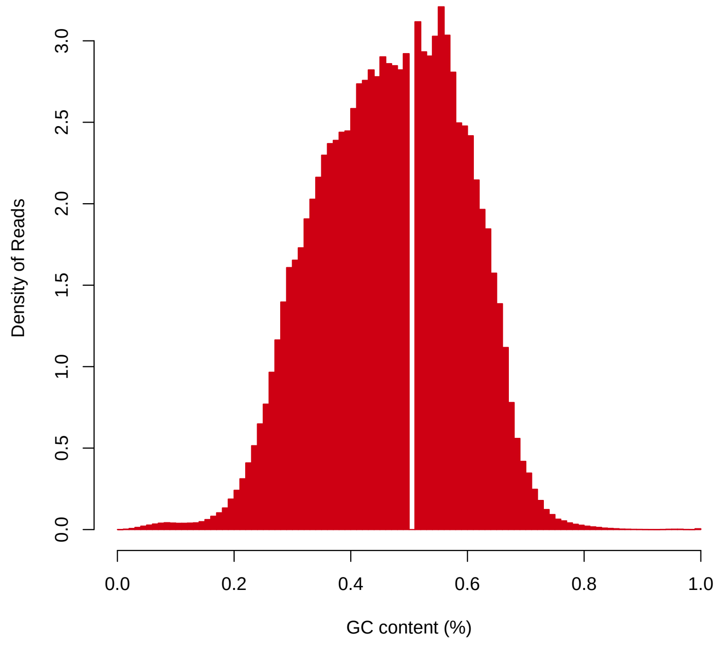

3 Bulk-cell level QC

In the bulk-cell level QC step we measured the performance of total Drop-seq reads. In this step we did't separate cell or remove "empty" cell barcodes, just like treated the sample as bulk RNA-seq sample.

3.1 Reads alignment summary

The following table shows mappability and distribution of total Drop-seq reads. It measures the general quality of data as a RNA-seq sample. Low mappability indicates poor sequence quality (see "Reads level QC") or library quality(caused by contaminant). High duplicate rate (low total UMI percentage observed, e.g. $< 10\%$) indicate insufficient RNA material and Overamplification. In summary, if the percentage of "total UMI count" is less than 5%, users may consider reconstruct your library (redo the experiment), but first you should make sure you already trim the adapter and map your reads to the correGetonded Getecies (genome version). Note that UMI number was calculated by removing duplicate reads (which have identical genomic location, cell barcode and UMI sequences). Mappable reads was after Q30 filtering if Q30 filter function was turned on.
* the percentage was calculated by dividing total reads number
** the percentage was calculated by divding total UMI number

Table 2: Reads alignment summary

genomic region (Category)	reads number
total reads	128,412,012
mappble reads	104,650,760 (81.5%)*
total UMI count	103,588,140 (80.67%)*
CDS exon UMI count	47,281,238 (45.64%)**
3'UTR UMI count	16,385,197 (15.82%)**
5'UTR UMI count	17,513,178 (16.91%)**
intron UMI count	13,776,376 (13.3%)**
intergenic UMI count	8,632,151 (8.33%)**

3.2 Gene body coverage

Aggregate plot of reads coverage on all genes. This module measures the general quality of the Drop-seq data. Theoretically we observe a unimodal (single bell) distribution, but for Drop-seq sample an enrichment at 3'end is observed due to library preparation using oligo-dT primers. In any case you should observe a smooth distritbuion. If loss of reads or Getike are observed in certain part of gene body (e.g. middle or 3'end of gene body), poor quality of your library was indicated. EGetecially when low mappability and high intron rate are also observed (see "Reads alignment summary" section).

Figure 4: Gene body coverage

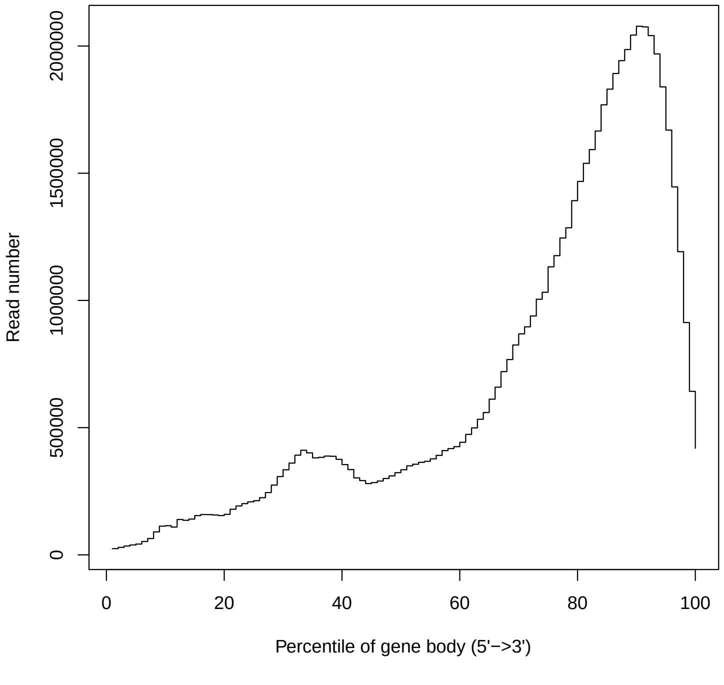

4 Individual-cell level QC

In this step we focused on the quality of individual cell and distinguishing cell barcodes from STAMPs (single-cell transcriptomes attached to microparticles)

4.1 Reads duplicate rate distribution

Drop-seq technology has an innate advantage of detecting duplicate reads and amplification bias due to the barcode and UMI information. This module diGetlays the distribution of duplicate rate in each cell barcode and helps to discard barcodes with low duplicate rate (which usually caused by empty cell barcodes and ambient RNA). We plot the distribution of duplicate rate in each cell barcode (though most of cell barcodes don't contain cells, they still have RNA) and observed a bimodal distribution of duplicate rate. We set an option for you to discard cell barcodes with low duplicate rate in following steps. The vertical line represented the cutoff (duplicate rate >= 0.1) of discarding cell barcodes with low duplicate rate. You can adjust the cutoff and rerun Dr.seq if current cutoff didn't separate two peaks from the distribution clearly (usually happened with insufficient sequencing depth). If the distribution didn't show clear bimodal or you don't want to discard cell barcodes according to duplicate rate, you can set cutoff to 0 to keep all cell barcodes for following steps.

Figure 5: Reads dupliate rate distribution

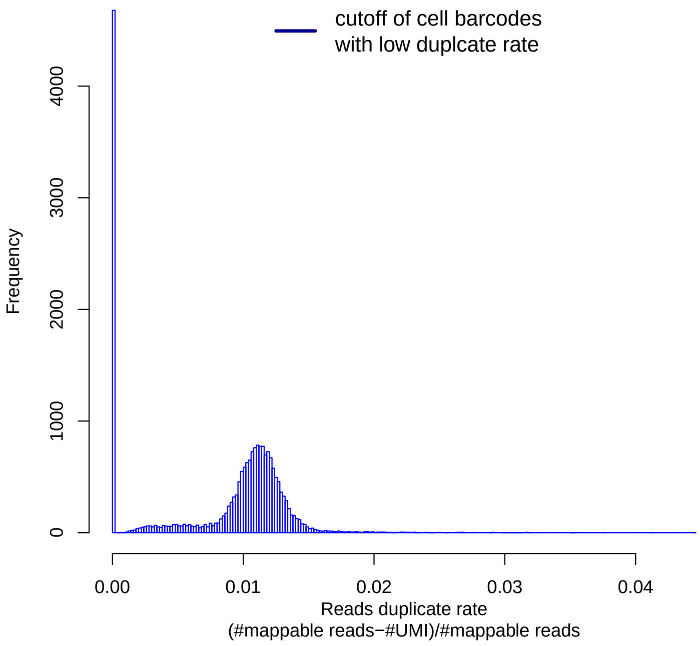

4.2 Reads duplicate rate vs. cumulative covered gene number

Reads duplicate rate versus cumulative covered gene numbers. This module measures whether each of your individual cell was sequenced and clearly separated from empty cell barcodes. Cell barcodes are ranked by the number of covered genes. The duplicate rate (y-axis, left side) is plotted as a function of ranked cell barcode. Red curve represents the number of genes covered by top N cell barcodes (y-axis, right side). N is diGetlayed by x-axis. Theoretically you observe a "knee" on your cumulative curve (slope = 1 on the curve) and the cutoff of your selected STAMPs (dash line) should be close to the "knee". The cutoff can also be far away from the "knee" in some cases because you input too many cells and have insufficient average sequencing depth, then you should adjust your cutoff (to the position you get enough STAMPs and sufficient reads count) and rerun Dr.seq. See the description of the paramter "select cell measure" in the Manual.

Figure 6: Reads duplicate rate vs. cumulative covered gene number

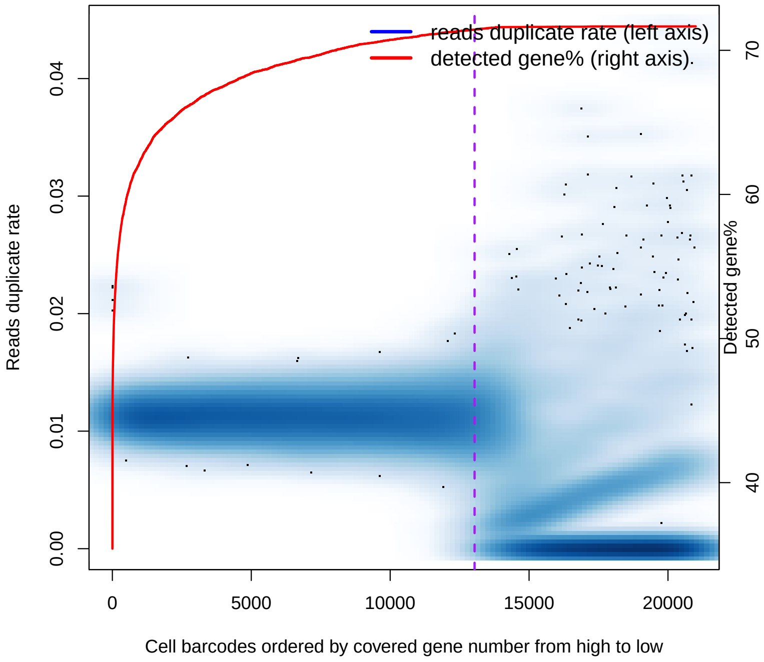

4.3 UMI vs. covered gene number

Covered gene number is plotted as a function of the number of UMI (i.e. unique read). This module measures the quality of Drop-seq experiment and helps to distinguish STAMPs from empty cell barcodes. We observe a clearly different pattern for two groups of cell barcodes with different reads duplicate rate (blue dots versus red and purple dots). Purple dots represented the selected STAMPs for the cell-clustering step. By default we select STAMPs with 1000 gene covered after discarding low duplicate cell barcodes. You may get few STAMPs according to this cutoff if the average sequencing depth of your cells was too low or too many cells were inputed. In this case you can adjust your cutoff or tell Dr.seq to directly select cell barcodes with highest reads count (see the description of the parameter "select cell measure"). Note that we use only STAMPs selected in this step for following analysis. The other cell barcodes are discarded.

Figure 7: UMI v.s. covered gene number

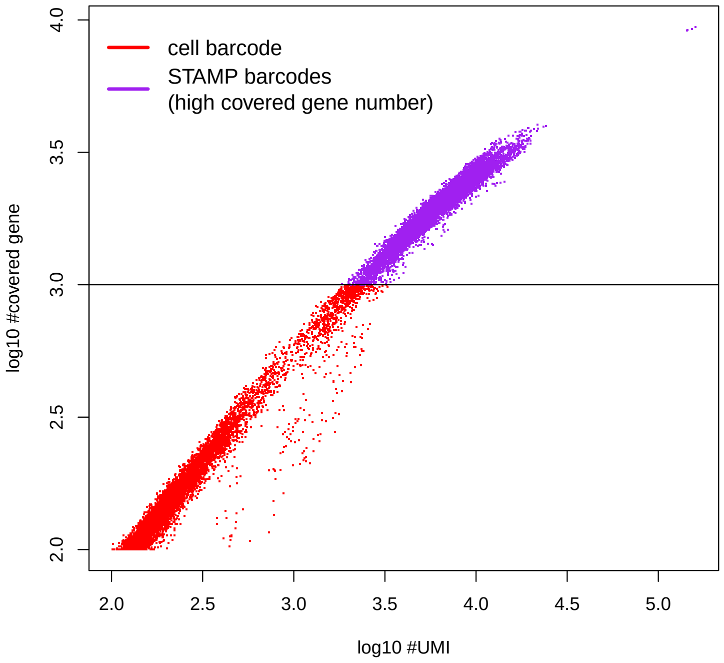

4.4 Covered gene number distribution

Histogram of covered gene number of selected STAMPs. The module measures whether the selected STAMPs have sufficient reads coverage. By default Dr.seq selects cell barcodes with >= 1000 genes covered as STAMPs. If you choose to select STAMPs with highest reads count ("select cell measure" = 2), then you should check this figure to make sure the STAMPs you select have enough gene covered. If most of your STAMPs have low covered gene number (e.g. < 100 gene covered), you can make your cutoff more stringent (e.g. select less cell barcodes with higher reads count) to make sure you get reliable STAMPs.

Figure 8: Covered gene number

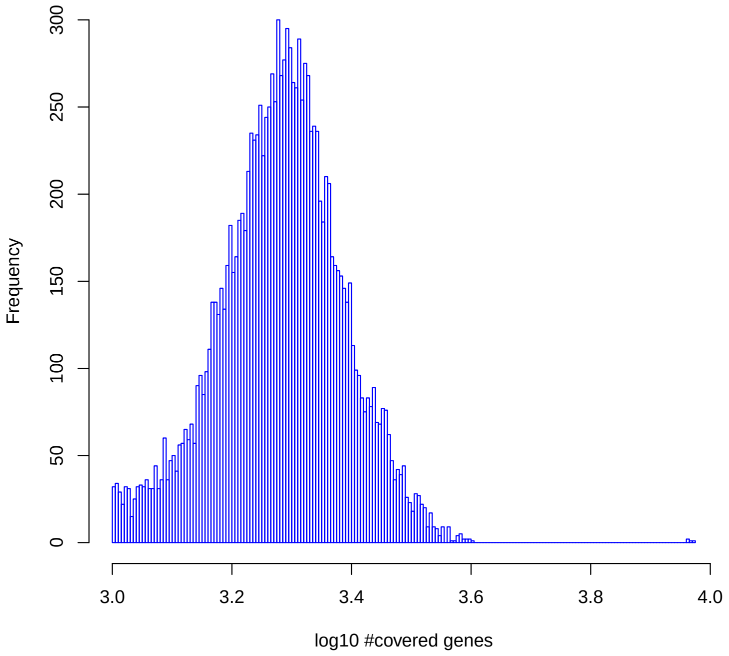

4.5 Intron rate distribution

Intron rate is a effective method to measure the quality of a RNA-seq sample. We plot a histogram of intron rate of every STAMP barcodes to check whether reads from each STAMPs enriched in the exon region. High intron rate (e.g. $>= 30\%$) indicates low quality of RNA in each STAMPs (caused by different problem, for example contaminant). You may consider your Drop-seq data low quality if most of selected STAMPs have high intron rate and low covered gene number (see "Covered gene number distribution" section). Intron rate is defined as $\frac{intron\ reads\ number}{intron+exon\ reads\ number}$

Figure 9: Intron rate distribution

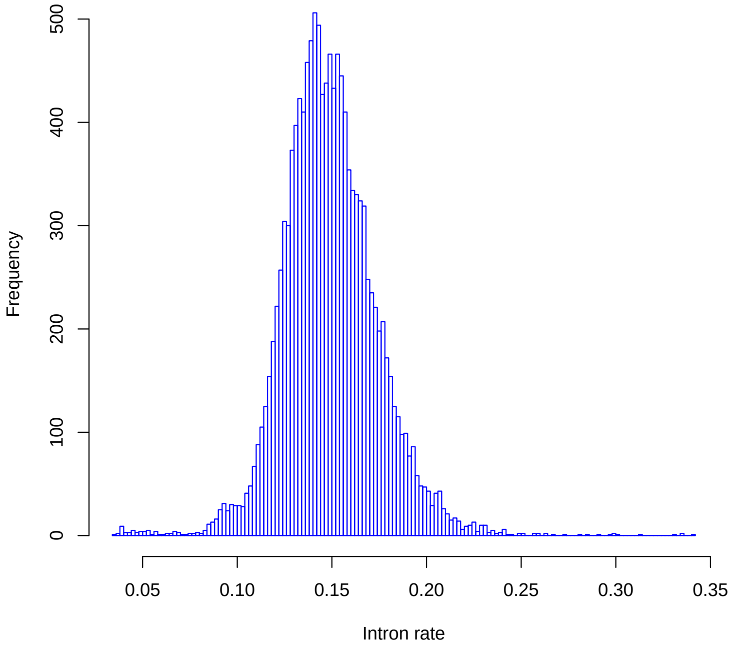

5 Cell-clustering level QC

This step composed by k-means clustering based on dimentional reduction result and Gap statistics to determine best k.

5.1 Gap statistics

We conducted a k-means clustering based on dimensional reduction output to measure sample's ability to be separated to different cell subtypes. Gap statistics was performed to determine the best k in k-means clustering. In general, decreasing pattern (usually k <= 2) is observed for pure cell type or cell line data, while increasing pattern with bigger k should be observed for mix cell types (or cell subtypes) data. If the cluster number predicted from the Gap statistics is largely different to what you expect, it indicated that your cells are not well characterized and separated by the Drop-seq experiment (due to the contaminant or the low capture efficiency of Droplets). In this case, you may consider your Drop-seq data poor quality. Alternatively, you may would like to use the parameter "custom k" to Getecify the cluster number.

Figure 10: Gap statistics

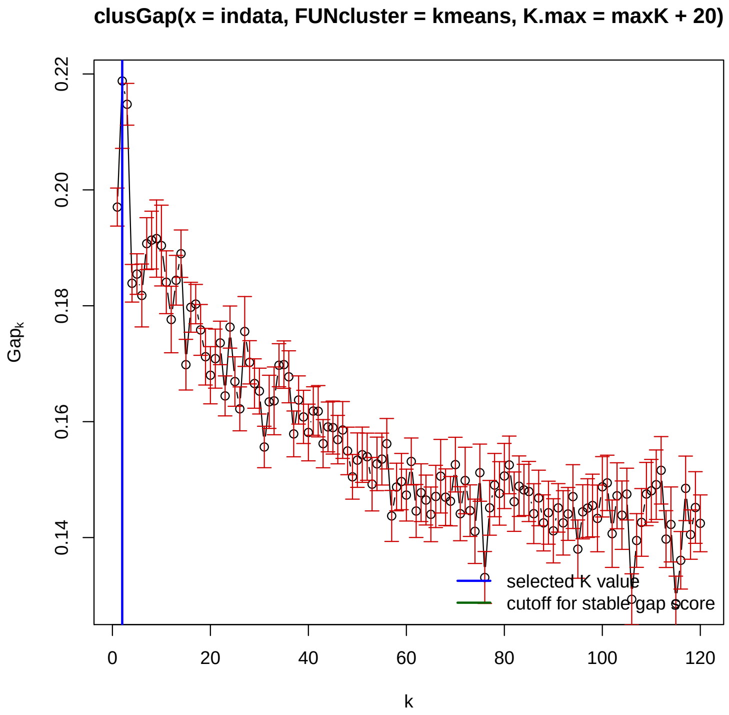

5.2 Clustering plot

Scatter plot represented visualization of dimensional reduction output of selected STAMP barcodes. STAMP barcodes are colored according to the clustering result and cluster numbers are printed in the center of each cluster. This figure is mainly for visualization and help you to know how your Drop-seq data look like. If you want to combine some small groups which are close to each other, you can use the cluster matrix (named "cluster.txt") in the Dr.seq standard analysis output to conduct your own analysis.

Figure 11: Clustering plot

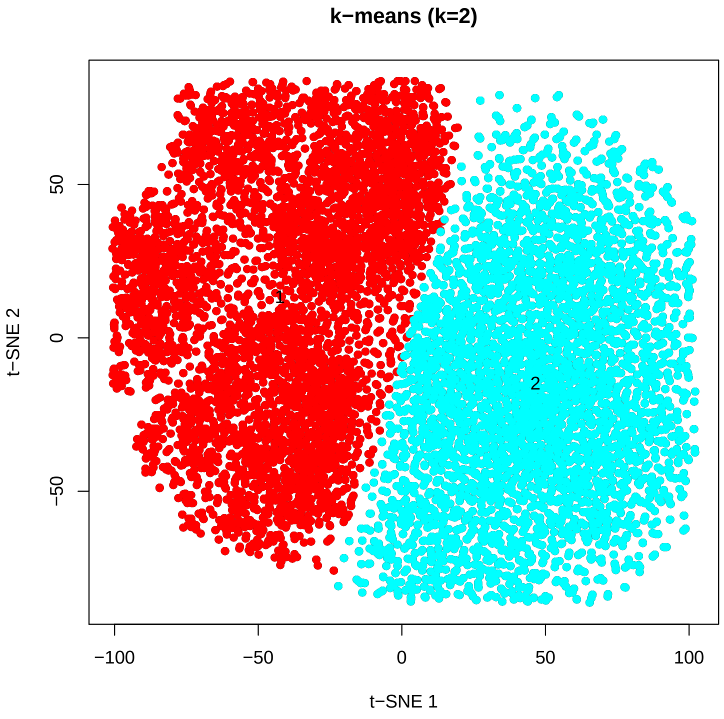

6 Output list

All output files were described in the following table

Table 3: output list

description	filename
expression matrix for selected STAMPs	jurkat_293t_50_50_expmat_clustercell.txt
top2 components of PCA dimentional reduction result	jurkat_293t_50_50_pctable.txt
pairwise correlation matrix	jurkat_293t_50_50_correlation_table.txt
All features of selected STAMPs	jurkat_293t_50_50_pctablefeatures_clustercell.txt
summary QC report	jurkat_293t_50_50_summary.pdf
